# Mid-Infrared Photothermal Spectroscopy for the Detection of Caffeine in Beverages

**DOI:** 10.3390/s24061974

**Published:** 2024-03-20

**Authors:** Giovanna Ricchiuti, Lisa Riedlsperger, Alicja Dabrowska, Erwin Rosenberg, Liam O’Faolain, Bernhard Lendl

**Affiliations:** 1 Institute of Chemical Technologies and Analytics, TU Wien, Getreidemarkt 9/164, 1060 Vienna, Austria; giovanna.ricchiuti@tuwien.ac.at (G.R.); lisa.riedlsperger@tuwien.ac.at (L.R.); alicja.dabrowska@tuwien.ac.at (A.D.);; 2 Centre for Advanced Photonics and Process Analysis, Munster Technological University, Bishopstown, T12 P928 Cork, Ireland; william.whelan-curtin@mtu.ie; 3 Tyndall National Institute, Lee Maltings, Dyke Parade, T12 R5CP Cork, Ireland

**Keywords:** mid-infrared spectroscopy, caffeine, gas chromatography, FTIR, photothermal spectroscopy, quantum cascade laser

## Abstract

Caffeine is the most widely consumed stimulant and is the subject of significant ongoing research and discussions due to its impact on human health. The industry’s need to comply with country-specific food and beverage regulations underscores the importance of monitoring caffeine levels in commercial products. In this study, we propose an alternative technique for caffeine analysis that relies on mid-infrared laser-based photothermal spectroscopy (PTS). PTS exploits the high-power output of the quantum cascade laser (QCL) sources to enhance the sensitivity of the mid-IR measurement. The laser-induced thermal gradient in the sample scales with the analytes’ absorption coefficient and concentration, thus allowing for both qualitative and quantitative assessment. We evaluated the performance of our experimental PTS spectrometer, incorporating a tunable QCL and a Mach–Zehnder interferometer, for detecting caffeine in coffee, black tea, and an energy drink. We calibrated the setup with caffeine standards (0.1–2.5 mg mL^−1^) and we benchmarked the setup’s capabilities against gas chromatography (GC) and Fourier-transform infrared (FTIR) spectroscopy. Quantitative results aligned with GC analysis, and limits of detection matched the research-grade FTIR spectrometer, indicating an excellent performance of our custom-made instrument. This method offers an alternative to established techniques, providing a platform for fast, sensitive, and non-destructive analysis without consumables as well as with high potential for miniaturization.

## 1. Introduction

Caffeine stands out as the most widely consumed psychoactive substance [1,2,3]. Naturally present in various plants and foods, it is commonly found in everyday beverages such as tea, coffee, and energy drinks. These non-alcoholic drinks have gained immense popularity for their ability to boost vitality and improve performance [1,4,5]. However, the impact of caffeine on human health remains a subject of ongoing discussion.

While caffeine has been associated with potential benefits such as weight loss, improved cognitive function, and increased alertness, it is also known to have adverse effects. These drawbacks include anxiety, depression, potential fertility issues, sleep disturbance, elevated sugar levels, and possible complications during pregnancy and breastfeeding [6]. The physiological consequences are highly dependent on the dosage, with thresholds varying based on factors like age, sex, and individual health condition. For this reason, specific regulations have been adopted in each country to define caffeine content and thresholds for commercially available products. The American Food and Drug Administration defines caffeine as both a food and a drug, recommending a maximum daily intake of 400 mg [7]. Similarly, the European Food Safety Authority has set comparable limits, providing estimates of average daily intakes among Member States based on age and health conditions [8]. For industries, it is thus crucial to monitor and adjust caffeine concentrations to ensure compliance with current food and beverage regulations.

In the literature, a wide range of contemporary analytical techniques are reported for identifying caffeine in commercial drinks. The most commonly employed methods include UV–visible spectrometry, high-performance liquid chromatography (HPLC), and gas chromatography (GC). UV–vis spectrophotometric analysis is recognized for its cost-effectiveness, straightforward procedure, and reliable accuracy and reproducibility [9]. It has limitations such as low sensitivity and selectivity, making it a less suitable choice for trace-level detection [10]. For high sensitivity and selectivity, it is recommended to use more advanced analytical instruments such as GC or HPLC, hyphenated to appropriate detectors. Despite their excellent performance, GC systems are limited to the separation of compounds of sufficient volatility and thermal stability. In particular, with gas chromatography there are issues with non-volatile constituents of caffeine-containing drinks such as sugars, amino acids, or polyphenols, which normally cannot be analyzed by GC without derivatization and therefore either require suitable clean-up or tend to contaminate the system and eventually reduce its performance. Furthermore, with the exception of thermal conductivity detectors, all GC detectors are destructive and sample recovery is not feasible [11]. The use of highly combustible hydrogen carrier gas to separate individual components and as a carburant source for a flame ionization detector (FID) requires careful handling. While HPLC can be considered a more suitable option for the analysis of complex mixtures, it oftentimes requires the use of complex mobile phases and precise pH adjustments, adding to the time and cost of the procedures [12]. The procedural steps in the sample preparation typically involve hot water extraction, filtration, and dilution. All chromatographic techniques have in common that a considerable amount of time, in the range of tens of minutes, is needed for completing the separation process and for readjusting the chromatography parameters like oven temperature or solvent composition, as required for subsequent sample analysis. 

In the meantime, Fourier-transform infrared (FTIR) spectroscopy has been proposed as an alternative method for the fast and non-destructive determination of caffeine [13,14]. Operating within the mid-IR range (400–4000 cm^−1^), this technique investigates the ro-vibrational molecular transitions in gases, liquids, and solids, providing molecular-specific information [15,16,17]. Analytical techniques based on mid-IR spectroscopy generally require minimal or no sample preparation, ensuring simplicity. This technology has been widely adopted in various application areas. The most common method for sample analysis is transmission spectroscopy, where analytical information is obtained by measuring the exponential decay of radiation intensity upon passing through an absorbing sample, following the Lambert–Beer law [13]. Unfortunately, since absorbance is a function of the analyte’s concentration and optical pathlength, sensitivity enhancement is achievable only by extending the optical pathlength, which is often limited by strong solvent absorption. An increase in sensitivity by enlarging the optical path is not a viable option in practice due to the limited power output of a conventional IR source (i.e., globar). Consequently, pathlengths of a few micrometers are commonly employed to prevent total IR absorption [14], mainly caused by the solvent used. This limitation results in a diminished sensitivity of measurements, leading to a less favorable limit of detection (*LOD*) and limit of quantification (*LOQ*).

These challenges can be addressed by the adoption of indirect measuring schemes. Among these schemes [18,19], photothermal spectroscopy (PTS) has proven to be a very sensitive method for detecting different molecules in gaseous and/or condensed-phase samples [20,21]. In this approach, the measurement does not rely on intensity changes upon IR absorption but instead focuses on the heat generated within the sample, altering its optical properties (i.e., refractive index). These changes can then be measured (probed) using various detection schemes, employing cost-efficient optics and more sensitive detectors operating in the visible or near-IR range. The magnitude of the thermal gradient photoinduced by the absorption of mid-IR photons corresponds to the sample’s absorption coefficient and concentration, thus reflecting the detectable PTS signal. Importantly, the generated indirect effect is not proportional to the pathlength, but scales linearly with the optical power of the mid-IR (pump) source. Hence, leveraging recent advancements in commercially available mid-IR quantum cascade laser (QCL) technology, which offers high optical powers of up to several hundreds of milliwatts [22], provides a way to improve the sensitivity. In addition, features such as room-temperature operation and compact packaging are favorable for the development of miniaturized and highly integrated PTS sensors serving various applications. 

To date, several detection schemes and configurations have been reported in the literature that utilize pump-probe PTS for analyzing samples in various forms [20,23,24,25,26,27,28]. We recently demonstrated a setup employing a tunable QCL and a Mach–Zehnder Interferometer (MZI) for detecting mid-IR absorption in liquid-phase samples. Our previous work demonstrated the setup’s applicability to trace-level water detection in organic solvents and aircraft jet fuel [29]. In this paper, our goal is to demonstrate the effectiveness of this technique for the measurement of the caffeine content in commonly consumed beverages, such as coffee, tea, and energy drinks. Our approach not only allows for the rapid monitoring of caffeine content and with minimal sample preparation, but potentially also provides additional insights into intermolecular interactions between the target analyte and the surrounding chemical environment. This encompasses both qualitative and quantitative information.

In this work, we comprehensively evaluate and validate the performance of our experimental PTS spectrometer for the measurement of the caffeine content in various beverages. This assessment includes external standards calibration, solid-phase extraction (SPE), and elution in chloroform as a preparatory step for commercial drink samples before analysis. Finally, we compare our results with GC and FTIR spectroscopy, serving as quantitative and qualitative reference methods, respectively.

## 2. Materials and Methods

### 2.1. Samples and Calibration Standards

Caffeinated samples of an energy drink (Red Bull Sugar Free, Salzburg, Austria), coffee (Jacobs, Bremen, Germany), and black tea (Teekanne Nero, Düsseldorf, Germany) were purchased from a local convenience store. In total, six standard solutions of caffeine were prepared by serial dilution in chloroform to concentrations of 0.1, 0.5, 1.0, 1.5, 2.0, and 2.5 mg mL^−1^. Caffeine anhydrous (99%) was purchased from Sigma Aldrich (Burlington, MA, USA). Chloroform (CHCl_3_), anhydrous (max. 0.005% H_2_O) and stabilized, was acquired from VWR Chemicals (Vienna, Austria). 

### 2.2. Solid Phase Extraction

SPE was employed for the extraction of caffeine and the removal of impurities from commercial drinks prior to their analysis. Disposable SPE columns, containing a polymerically bonded octadecyl reversed-phase sorbent (Discovery DSC-18 SPE tube, bed weight 500 mg, volume 6 mL from Supelco, Vienna, Austria), were arranged in a manifold and conditioned by passing 5 mL of CHCl_3_ and 5 mL of water. The aqueous samples of coffee, tea, and energy drink (each with volumes of 4 mL and 8 mL) were cooled and loaded onto the sorbent material. After passing through the columns at a flow rate of 0.2–0.5 mL min^−1^, the sorbent was rinsed with 1 mL of water and dried using a nitrogen stream. Subsequently, the analyte was eluted with 4 mL of CHCl_3_, and the resulting samples (sample 1—1:1 dilution; sample 2—2:1 dilution) were collected for further analysis.

### 2.3. Gas Chromatography

Quantitative GC analyses were carried out using a 2010 Plus gas chromatograph equipped with a flame ionization detector (FID) and an autosampler (all from Shimadzu, Kyoto, Japan). FID was chosen as the detector due to its sensitivity, linearity, and robustness. The separation column used in the analysis was a Restek RTX-5MS capillary column with 30 m length, 0.25 mm I.D., and a 0.25 μm film thickness (obtained from BGB Analytik, Rheinfelden, Switzerland). The parameters of the GC are summarized in Table 1. First, the baseline (chloroform) was measured, followed by the measurement of caffeine standards. Subsequently, the commercial drink samples were analyzed. The chromatograms were evaluated using the Shimadzu LabSolutions Single GC SW v.4.75 (Shimadzu, Kyoto, Japan).

### 2.4. FTIR Spectroscopy

A Bruker Vertex 80v FTIR spectrometer (Ettlingen, Germany), equipped with a globar and a liquid-N_2_-cooled MCT detector (D*= 4.0∙10^10^ cm Hz^1/2^ W^−1^ at 9.2 µm), was employed for all FTIR measurements. Samples were manually injected into a transmission flow cell, which featured two 2 mm thick CaF_2_ windows and a 330 μm thick Teflon spacer. A total of 200 scans were averaged per spectrum, resulting in an acquisition time of ~90 s. Spectra were recorded with a resolution of 2 cm^−1^ in double-sided acquisition mode and were calculated using a Blackman–Harris 3-term apodization function and zero filling factor of 2. During measurements, the spectrometer was purged with dry air for several minutes prior to data acquisition, ensuring consistent and minimized water vapor absorption. All measurements were performed at ambient temperature (23 °C). The OPUS 8.5.29 software package from Bruker (Ettlingen, Germany) was used for the evaluation of the recorded spectra. Water vapor absorption bands were subtracted if necessary.

### 2.5. Photothermal Spectrometer Based on a Mach–Zehnder Interferometer

The schematic and operational principles of our experimental photothermal spectrometer were described in detail in our previous works [29,30]. Nevertheless, a concise overview of the setup is hereby presented to aid the reader in understanding our detection and readout scheme. 

**Figure 1:**
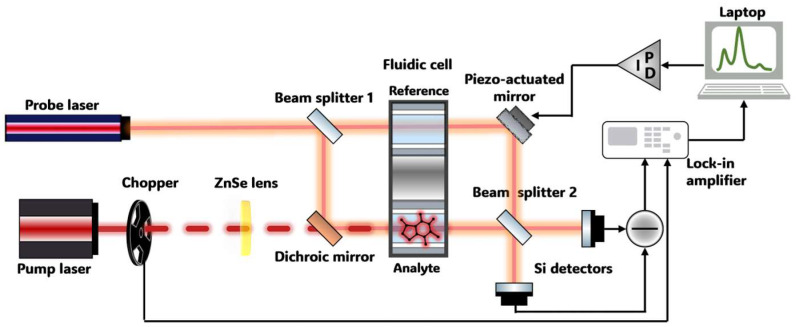
Schematic of an experimental pump-probe photothermal spectrometer for liquid-phase analysis based on a Mach–Zehnder interferometer.

Figure 1 illustrates the experimental spectrometer, utilizing pump-probe photothermal interferometry with a Mach–Zehnder-type interferometer. In this approach, a probe laser beam interacts with the sample, and the resulting changes in the thermal properties of a sample, directly proportional to its concentration, generate a modulation in the refractive index. This modulation, induced by the periodic heating and cooling of the sample, results in variations in the optical path length of the probe beam, which can be determined by means of interferometry.

A commercially available external cavity quantum cascade laser (EC-QCL) (DRS Daylight Solutions Inc., SanDiego, CA, USA), tunable between 1570 and 1725 cm^−1^, was used as an excitation (pump) source. The tunability region overlapped with the two absorption bands of caffeine, with peaks centered around 1660 and 1700 cm^−1^ [31]. The tunable laser was operated in CW mode with a laser current of 650 mA, resulting in an output power of 230 mW. The laser was tuned with a scan rate of ~50 cm^−1^ s^−1^ (~3 s scan). The operating temperature of the water-cooled laser head was set to 20 °C. The excitation beam was modulated by an external optical chopper at a frequency of 30 Hz and a duty cycle of 50%. These parameters were selected to maximize the photothermal response, inversely scaling with the modulation frequency, and to optimize the signal-to-noise ratio (SNR).

A linearly polarized He−Ne laser (Melles Griot, Rochester, NY, USA) emitting at 632.8 nm was coupled to a benchtop MZI and served as a probe laser. As per the Mach–Zehnder interferometric scheme, the probe beam was split into two beams via a 50:50 silica beam splitter. The two identical arms represented the reference branch (i.e., solvent solution) and the analyte branch (i.e., analyte + solvent solution). Following additional reflection from the ZnSe dichroic mirror (Laser Components, Olching, Germany), the two probe beams reached a dual-channel liquid transmission cell with a 110 µm Teflon spacer defining the analytical pathlength. In these cells, reference and sample solutions were injected, and the measurements were conducted in stopped flow mode. The mid-IR excitation laser was collinearly aligned to the analyte arm of the interferometer by means of a dichroic mirror, which transmitted the IR radiation and reflected the visible probe beam, thus acting as a beam combiner. Following the interaction with the sample at the output of the cell, a second beam splitter and a mirror were used to redirect the two interfering beams to the two silicon detectors (DET10A, Thorlabs, Newton, NJ, USA). 

The differential interference signal from the two detectors was directed to a lock-in amplifier (MFLI, Zurich Instruments), which demodulated the raw PTS signal using the optical chopper modulation frequency as a reference. The demodulated signal contained three pieces of information: (i) the power emission profile of the excitation laser, (ii) absorption from the solvent, and (iii) absorption from the analyte. To extract analytical information related only to the target analyte and thereby qualitatively reconstruct its spectrum, laser power normalization and background correction was performed in post-processing operations. Spectra were smoothed using a Savitzky–Golay filter (order: 3, window: 15 points/~14 cm^−1^). Further details on the software, signal acquisition, and data processing procedures employed are reported in the “Supplementary Materials”, where a step-by-step analysis is described and depicted. It is worth noting that the entire setup, as well as the liquid cell, were temperature-stabilized to minimize the impact of thermal changes from the external environment. This approach ensured that the detected phase shift was solely attributed to the thermal gradient induced by the IR absorption of the analyte present in the sample.

### 2.6. PTS Spectrometer Performance Assessment

Figures of merit and relevant metrics for the assessment of the PTS experimental spectrometer were determined following applied chemistry standards. In particular, the limit of detection (*LOD*) and limit of quantification (*LOQ*) were calculated by considering the standard deviation (std) *σ* of 30 replicates of the blank (CHCl_3_) recorded at zero/near zero concentration and the slope S of the calibration curve that represents the sensitivity as per ref. [32,33]:

(1) $$LOD=\frac{3\;\sigma }{S}$$

(2) $$LOQ=\frac{10\;\sigma }{S}$$

Moreover, by considering GC as a reference method for the quantitative evaluation of caffeine concentration in real samples, the root mean square error (*RMSE*) was additionally computed. *RMSE* represents the gap between the predicted and actual values (residuals). Assuming the GC measured concentrations as actual values and PTS measured ones as predicted, *RMSE* was evaluated as:

(3) $$RMSE=\sqrt{\frac{1}{n}\sum_{i=1}^{n}{\left(x_{PTS}-x_{GC}\right)}^{2}}$$

where n is the number of commercial drink samples (n=6). *LOD*s, *LOQ*s, and computed values of the *RMSE* are reported in the following “Results and Discussion” subsections. 

## 3. Results and Discussion

In this section, quantitative results of GC analysis are presented first, followed by the FTIR analysis of the caffeine standards. Subsequently, the mid-IR photothermal spectra of caffeine in both standards and beverage samples will be presented. Detailed information on the corresponding performance metrics and the derived caffeine content will be given, along with a discussion on the recorded FTIR and PTS spectra. 

### 3.1. Quantitative Analysis—Gas Chromatography

Gas chromatography analyses were performed for (i) pure chloroform, (ii) caffeine calibration standards, and for (iii) commercial drink samples (two volumes for each sample type). The FID utilized for the analyses measured the conductivity of an oxyhydrogen flame. The components targeted for analysis were separated and sequentially introduced into the flame, where thermal ionization and combustion occurred. The electrons released during ionization were collected by a ring-shaped grid anode. The resulting current is proportional to the number of oxidizable carbon atoms eluting per second. Additionally, the signal is linearly proportional to the carbon content of the analyte across a broad concentration range.

**Figure 2:**
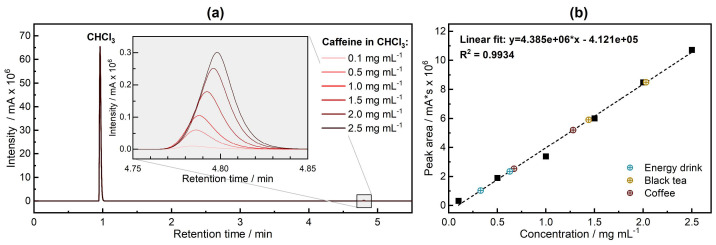
(**a**) Chromatogram of standard solutions of caffeine. The inset shows intensity peaks for different concentrations of caffeine. (**b**) Calibration line for the six calibration standards (black squares) and retrieved caffeine concentrations in the commercial drink samples (colored circles).

The results of the GC analysis for the caffeine standards are presented in Figure 2. Both the standards and the blank chromatograms exhibit a prominent peak at around 0.96 min, which is attributed to the chloroform solvent. The chromatogram of standards and samples exhibited a peak at a retention time of approx. 4.8 min, corresponding to caffeine (see inset in Figure 2a). As per Table 1, a total time of 11 min is needed for sample analysis, including oven ramping and retention time. Additionally, time must be allowed for the instrument to cool down and equilibrate at start conditions between one injection and the next (~2–3 min). This leads to an overall ~15 min time per each sample injection and analysis. By plotting the peak areas against the caffeine concentrations of the standards, a calibration line is derived, as illustrated in Figure 2b.

Following the measurements of the standards, two coffee samples, two black tea samples, and two energy drink samples were measured. Utilizing the linear fitting equation, the concentrations for the real samples were determined. The results are gathered in Table 2.

### 3.2. Qualitative Analysis—FTIR Spectroscopy

**Figure 3:**
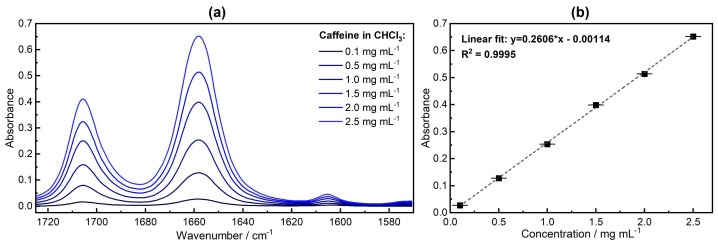
(**a**) FTIR absorption spectra of the caffeine standard solutions. (**b**) Calibration line for the six calibration standards. Quantitative evaluation was performed with the band maximum at ~1660 cm^−1^.

Unlike GC, FTIR spectroscopy provides fast, non-destructive analysis, and in addition to quantitative data, it offers qualitative insights into the samples. Figure 3a shows the measured FTIR spectra of caffeine standard solutions. Caffeine demonstrated two primary absorption peaks, at ~1700 cm^−1^ and ~1660 cm^−1^, corresponding to the two carbonyl groups, coupled into an in-phase (C=O)_2_ stretching vibration and an out-of-phase (C=O)_2_ stretching vibration, respectively [32]. A minor band at 1605 cm^−1^ arose from residual water in chloroform. This finding is consistent with observations reported in our previous work, where the impact of different organic solvents on the bending vibration of water was discussed [29].

Furthermore, the recorded spectra were assessed quantitatively. The calibration curve and uncertainties are presented in Figure 3b. The noise level of the 100% lines of chloroform was calculated in the spectral region between 1570 and 1725 cm^−1^ and found to be 1.9 × 10^−4^ (average of 10 measurements), whereas the *LOD* and *LOQ* were 0.0022 mg mL^−1^ and 0.0073 mg mL^−1^, respectively. Please refer to the “Supplementary Materials” for the methodology adopted for uncertainties computation.

### 3.3. Qualitative and Quantitative Analysis—PTS Spectroscopy

**Figure 4:**
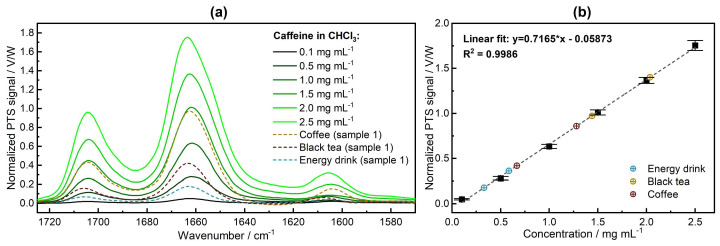
(**a**) Mid-IR photothermal spectra of the caffeine standard solutions and commercial drink samples. (**b**) Calibration line for the six calibration standards (black squares) and retrieved caffeine concentrations in the commercial drink samples (colored circles). Quantitative evaluation was performed with the band maximum at ~1660 cm^−1^.

The experimental MZI-based PTS spectrometer was used to record mid-IR photothermal spectra of the samples containing caffeine (see Figure 4). The spectrometer was first calibrated with the standard solutions, and then the commercial drink samples were measured. For the optimum SNR previously determined in our work [29], 30 scans were averaged per spectrum, resulting in a total acquisition time of 90 s per sample. 

As for the comparison of the qualitative results, the band shape and position of absorption peaks were in good agreement with the reference FTIR method. However, in the PTS technique, the thermal properties of the sample’s constituents play a crucial role in the strength of the PTS signal. For example, the water contribution at around 1605 cm^−1^ appears slightly stronger. This is attributed to the fact that the significantly higher absorption coefficient of water compared to chloroform in this wavenumber range results in a more pronounced absorption of the excitation laser power. On the other hand, as previously demonstrated in our studies [29,30], the chemical environment surrounding the target analyte can either enhance or diminish the photothermal signal from the sample, which in this case consists of caffeine but also small contaminations of co-extracted water still present after SPE. As a solvent, chloroform possesses a high thermo-optical coefficient (dn/dT) of −6.03 × 10^4^ K^−1^ [20], implying a higher PTS signal amplitude.

In the next step, the acquired PTS spectra were assessed quantitatively for the band maximum at ~1660 cm^−1^. A calibration curve for a range of standard caffeine solutions was obtained and its linear fitting parameters were used to determine the concentration of caffeine from an extract of commercial drinks. The determination of uncertainties reported in Figure 4b is described in the “Supplementary Material”. For illustration, spectra of the extracts (Sample 1) are reported in Figure 4a. The quantitative results are gathered in Table 3. 

The noise level, as well as the *LOD* and *LOQ*, were evaluated and compared to the FTIR instrument. The noise level was found to be 3.6 × 10^−4^ *V*/*W*, whereas the *LOD* and *LOQ* were determined to be 0.0015 mg mL^−1^ and 0.0051 mg mL^−1^, respectively. The sensitivity of our PTS spectrometer is thus comparable with that of a high-end FTIR instrument.

**Figure 5:**
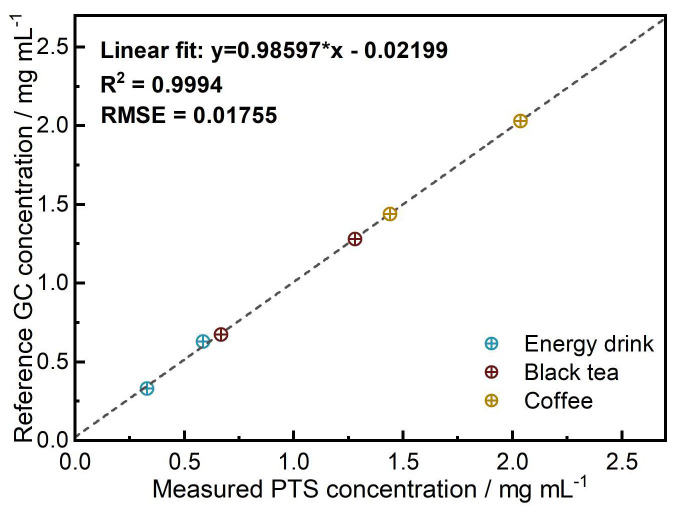
Correlation between caffeine concentrations in commercial drinks determined by reference GC ($x_{GC}$) and PTS spectroscopy ($x_{PTS}$).

The quantitative results obtained for commercial drink samples are in excellent agreement with the reference GC method, with a root mean square error (*RMSE*) of 0.018 mg mL^−1^, highlighting the high accuracy of our PTS spectrometer (see Figure 5). 

## 4. Conclusions

In this work, we proposed an alternative technique to gas chromatography and FTIR spectroscopy for comprehensive caffeine content analysis based on mid-IR photothermal spectroscopy. 

In contrast to GC, our technique offers several key advantages, including (i) minimal acquisition time, down to 3 s per individual scan and sample injection (~100 samples/h), as opposed to ~15 min (4 samples/h); (ii) the capability to provide information on the intermolecular interactions within the studied mixture; and (iii) direct and label-free analysis, allowing for the potential development of an online/inline miniaturized sensor version. The results show that very good agreement between the caffeine concentrations in beverage samples was obtained from the two analysis methods, confirming that our proposed method is a valid option to adopt.

When compared to FTIR spectroscopy, our approach demonstrates comparable *LOD* and *LOQ*, and the obtained spectra show excellent agreement. Whereas a liquid-nitrogen-cooled detector had to be used for reaching the required sensitivity with an FTIR spectrometer, the PTS spectrometer is operated at room temperature and reaches comparable sensitivities. Additionally, in the FTIR measurement, the analytical pathlength was three times longer. Even though the analytical pathlength influences the strength of the PTS signal, the choice of a smaller pathlength in our PTS spectrometer was made to remain within the limited dynamic range of the visible detectors used for the probe for the investigated concentration range [29]. Improvements in performance and subsequent figures of merit could thus be achieved using different detectors, but also by using different modulation frequencies or employing active quadrature point control. Nevertheless, the output power of the laser remains a more influential parameter in determining sensitivity in the proposed technique. Therefore, adopting a newer generation EC-QCL with higher optical powers and a faster, more accurate tuning mechanism for enhanced scan-to-scan alignment could further diminish noise levels and improve overall performance. This emphasizes the critical role of evolving technology in shaping the effectiveness of the technique. 

From a cost perspective, as already mentioned, PTS mainly relies on harnessing the robust emission capabilities of QCLs to excite the sample, which constitutes the main expense in our spectrometer. In fact, the full interferometer is built using a HeNe probe laser along with cost-effective optics and sensitive detectors within the visible spectrum range, in contrast to the costly components needed for the mid-IR region used in the FTIR spectrometer. When considering the GC costs, one must consider carrier gas tank and column replacements as a periodical expense throughout the whole lifetime of the instrument, based on the usage. Additionally, regular maintenance services must be performed for the FID. Over time, samples start to contaminate the system, leading to a loss of sensitivity, high baselines, noise peaks, and/or flame non-ignition.

Currently, companies in the beverage and food industry that manufacture caffeinated products must adhere to quality assessment standards outlined in ISO-20481 [33]. This involves employing an extraction process followed by HPLC analysis to determine the caffeine content, which is an off-line method. With ongoing technological progress, the feasibility of developing compact and portable mid-IR PTS devices utilizing QCLs and photonic integrated circuits (PICs) for the detection of refractive index changes in liquids can allow the further development of QCL-based PTS sensors, for on-line as well as in-line monitoring applications [34,35,36]. As an illustration, incorporating an autosampler into the industrial production process enables the extraction of a small sample, facilitating rapid analysis. This approach allows the liquid to be directed to microfluidics, situated on waveguides, to be analyzed much more quickly compared to conventional methods. No reagents are required, and if the probe laser is selected appropriately, PTS allows for the use of water as a matrix, a possibility that is not feasible in GC.

## Figures and Tables

**Table 1:** Operational parameters of the gas chromatograph.

Parameter	Description
Injection port	EPC (electronic pressure control)-controlled split/splitless inlet operating in split mode
Injection volume	1 µL
Carrier gas	Helium
Inlet parameters	Heater temperature: 250 °C, gas pressure: 29.4 psi, total flow 129 mL min^−1^, split ratio: 1:50, split flow: 125 mL min^−1^
Column flow	2.5 mL min^−1^ (constant flow), 52 cm s^−1^ velocity
Detector temperature	300 °C
GC oven program	120 °C for 1 min hold time, then ramped at a rate of 20 °C min^−1^ to 240 °C, then ramped at a rate of 30 °C min^−1^ to 270 °C for 3 min hold time (total time of 11 min)

**Table 2:** Caffeine content in commercial drinks (in mg mL^−1^) determined by gas chromatography.

Coffee		Black Tea		Energy Drink	
Sample 1	Sample 2	Sample 1	Sample 2	Sample 1	Sample 2
1.44	2.03	0.67	1.28	0.33	0.63

**Table 3:** Caffeine content in commercial drinks (in mg mL^−1^) determined by PTS spectroscopy.

Coffee		Black Tea		Energy Drink	
Sample 1	Sample 2	Sample 1	Sample 2	Sample 1	Sample 2
1.44	2.04	0.67	1.28	0.33	0.59

## Data Availability

Data available within the article. Raw data will be made available upon request.

## Supplementary Materials

The following supporting information can be downloaded at: https://www.mdpi.com/article/10.3390/s24061974/s1.
