# Supplementary material for: Mid-Infrared Photothermal Spectroscopy for the Detection of Caffeine in Beverages
Source: Sensors (Basel). 2024 Mar 20;24(6):1974. doi: 10.3390/s24061974 (PMC10975067; doi:10.3390/s24061974)
Supplement: Supplementary file 1 [file sensors-24-01974-s001.zip › sensors-2892092-supplementary.pdf]

SUPPLEMENTARY MATERIAL

Data acquisition and processing

Lock-in Amplifier (LIA) raw signal [V] consisted in 30 scans per blank ($CHCl_3$) and per each sample concentration. Water vapor contribution is a high source of noise in the mid-IR spectral region of interest (1725-1570 cm^{-1}).

The raw data from LIA were recorded by means of a data acquisition card from National Instruments (NI), (DAQ Card, 6001-USB, National Instruments, Salzburg, Austria), USB-connected to a personal computer. A custom-made developed LabVIEW (licensed) GUI was used to create a real-time .txt file containing the LIA demodulated signal synchronized with the forward-scan trigger of the EC-QCL. This way the data recorded while the grating goes backward are not saved. Each scan is constituted by 167 datapoints. This .txt file is opened and processed using Matlab R2022a – academic use (licensed).

The so-called normalized PTS signal is calculated starting from the raw recorded data as:

$$Normalized\ PTS\ Signal = \left(\frac{I_{c\left[\frac{mg}{mL}\right]}[V] \cdot 1000\ - I_{blank}\ [V] \cdot 1000}{P(\tilde{\nu})[mW]} \right)$$

30 scans were recorded for the blank and for the 6 external standards.

The 30 scans were then averaged, background corrected and further normalized by the optical power of the EC-QCL which is wavelength dependent. A final smoothing was performed to remove small, remained noise features for better comparison against high-end FTIR spectrometer qualitative spectra.

The original data are reported in the following as well as a description of the methodology used for retrieving the Normalized PTS spectra reported in the main manuscript (see Figure 4a and 4b).

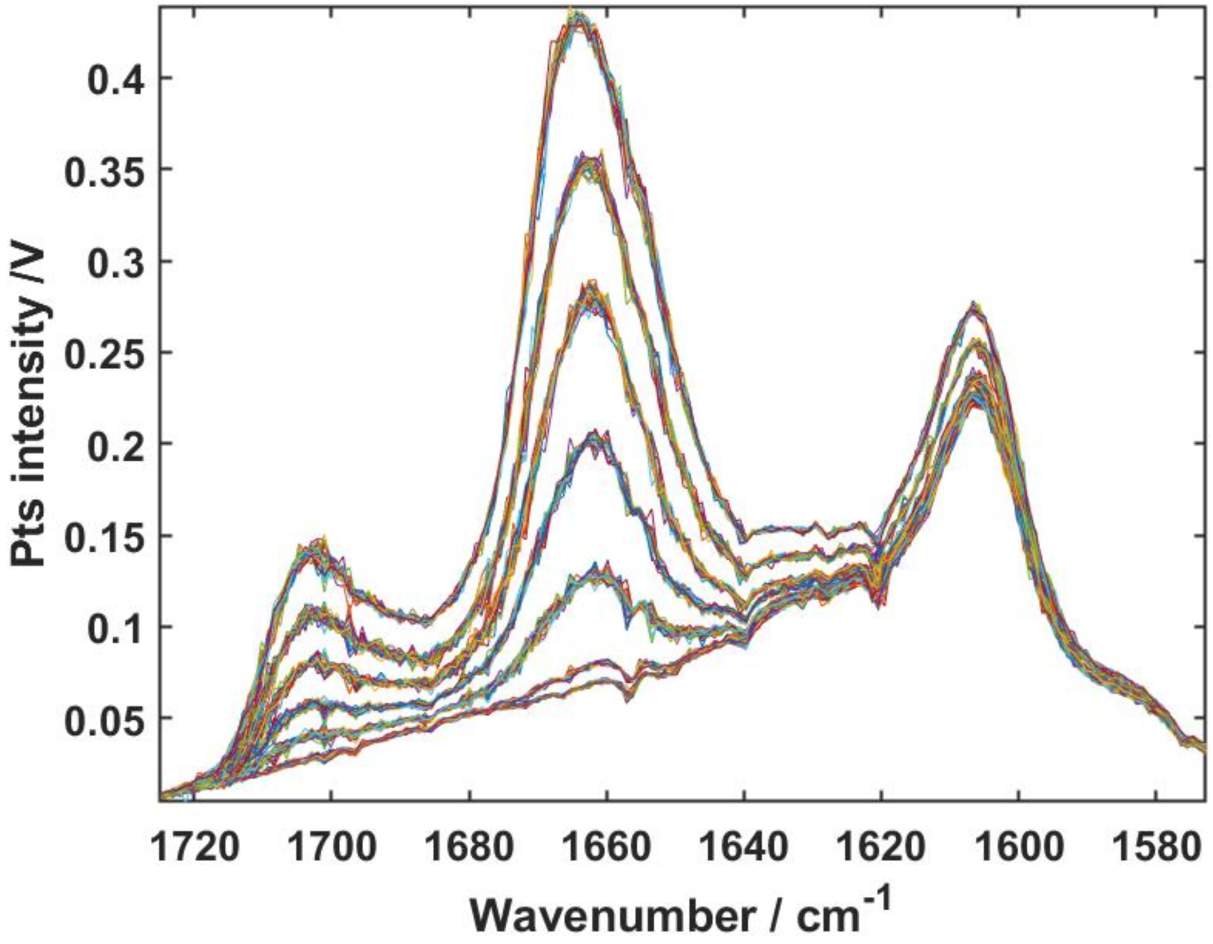

Figure S1. Raw LIA demodulated PTS signal for blank and external standards of caffeine in $CHCl_3$ samples.

30 scans were averaged:

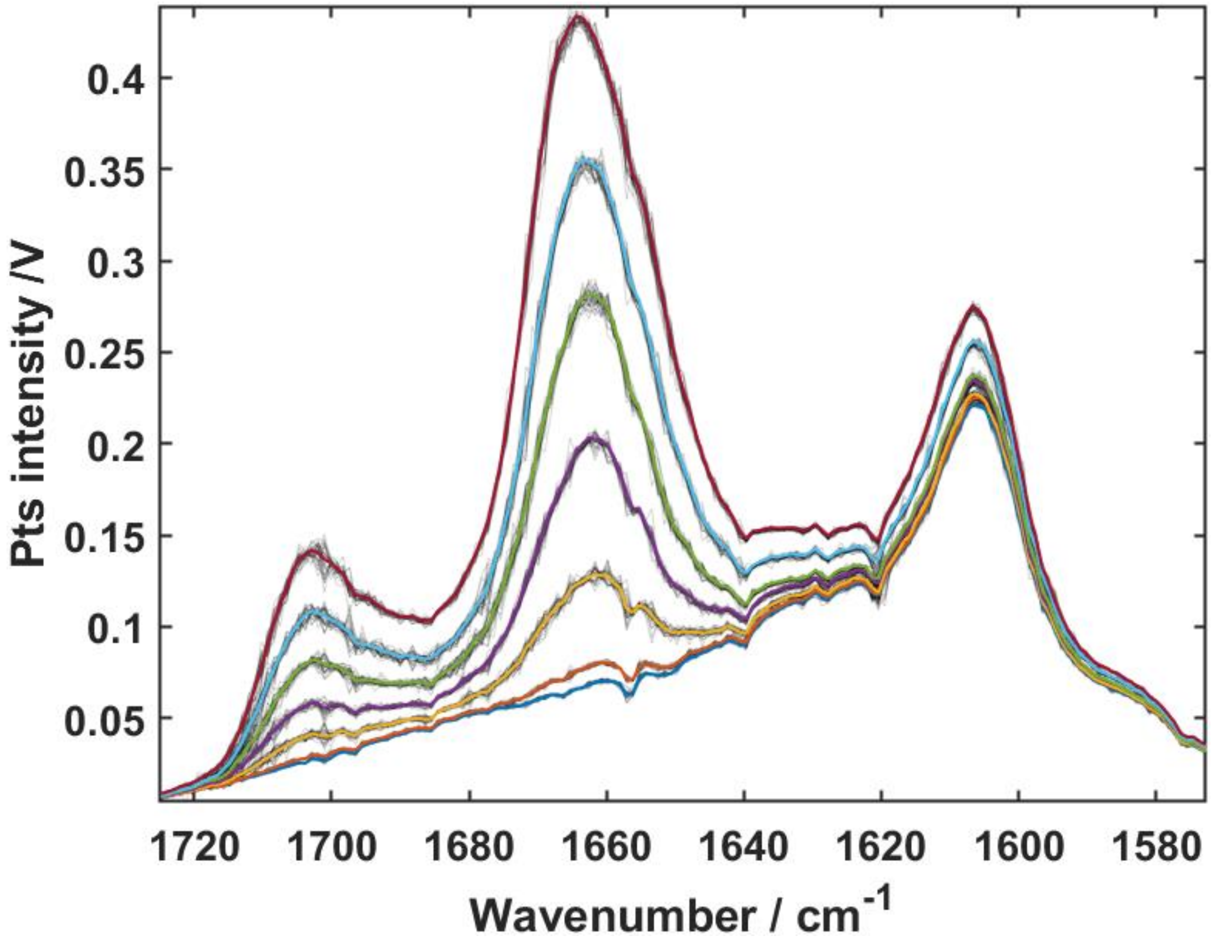

Figure S2. 30 scans averages per each sample and solvent. In gray opaque, the raw data (30 scans) trend is kept.

The spectra were corrected by the blank acquisition (background):

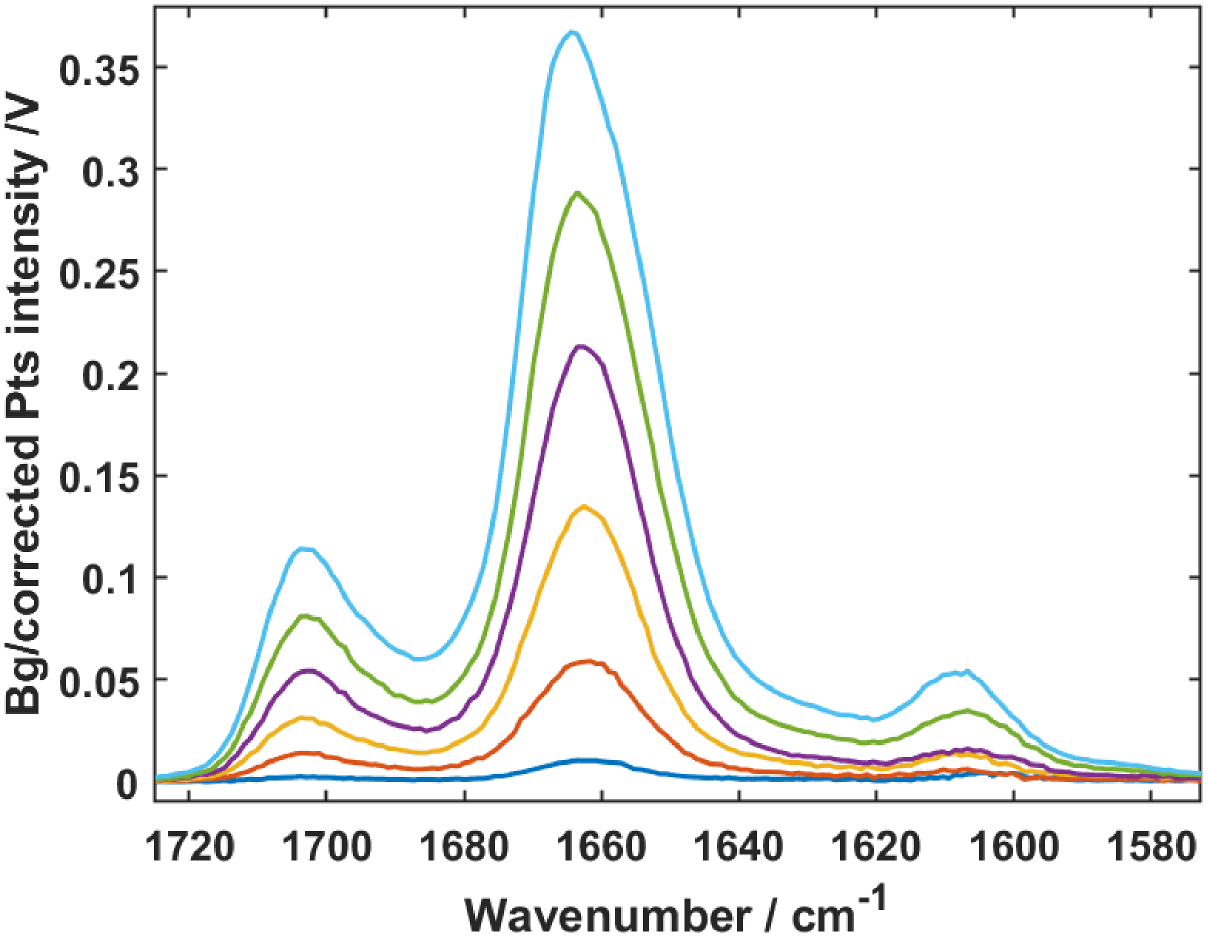

Figure S3. Unfiltered background-corrected spectra per each sample concentration.

In PTS, the signal is proportional to the optical power $P(\tilde{\nu})$ of the excitation laser, which is wavelength dependent. In such a technique, the background-corrected signals are further normalized by the mid-IR laser emission profile. In our setup, we do not record the optical power simultaneously while performing a measurement. The power spectrum was prior recorded by means of a power meter. Thus, the same spectrum is used to normalize the above-reported records.

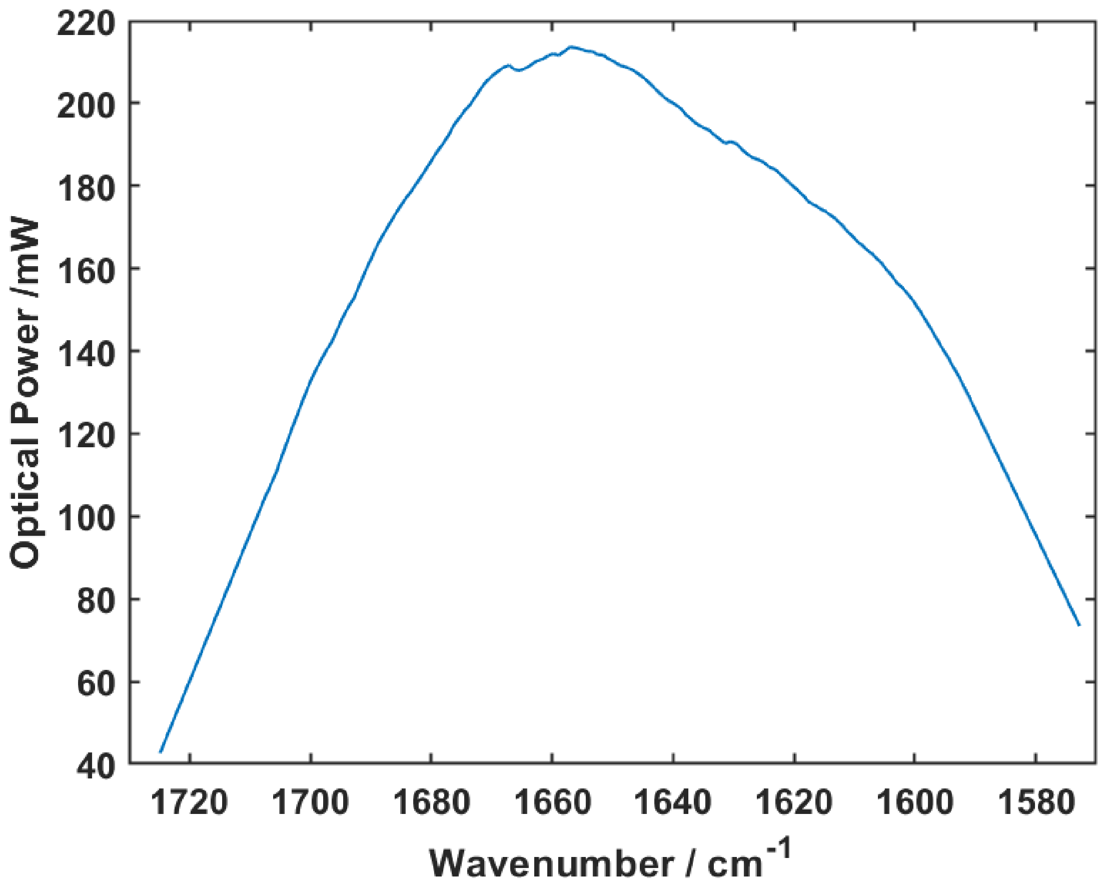

Figure S4. EC-QCL optical power spectrum recorded with power meter.

Since the recorded BG-removed PTS signal is in [V] and the optical power is in [mW]. We multiplied the BG-removed PTS signal in [V] by 1000. This way we obtain a resulting signal that is expressed in [V/W]. The same operation could be done by using an MCT detector, this way the ratio would become dimensionless. Ideally the optical power should be recorded simultaneously to correct small thermal change and environmental conditions (e.g. humidity percentage).

The spectra, multiplied by 1000 and divided by the optical power are reported below:

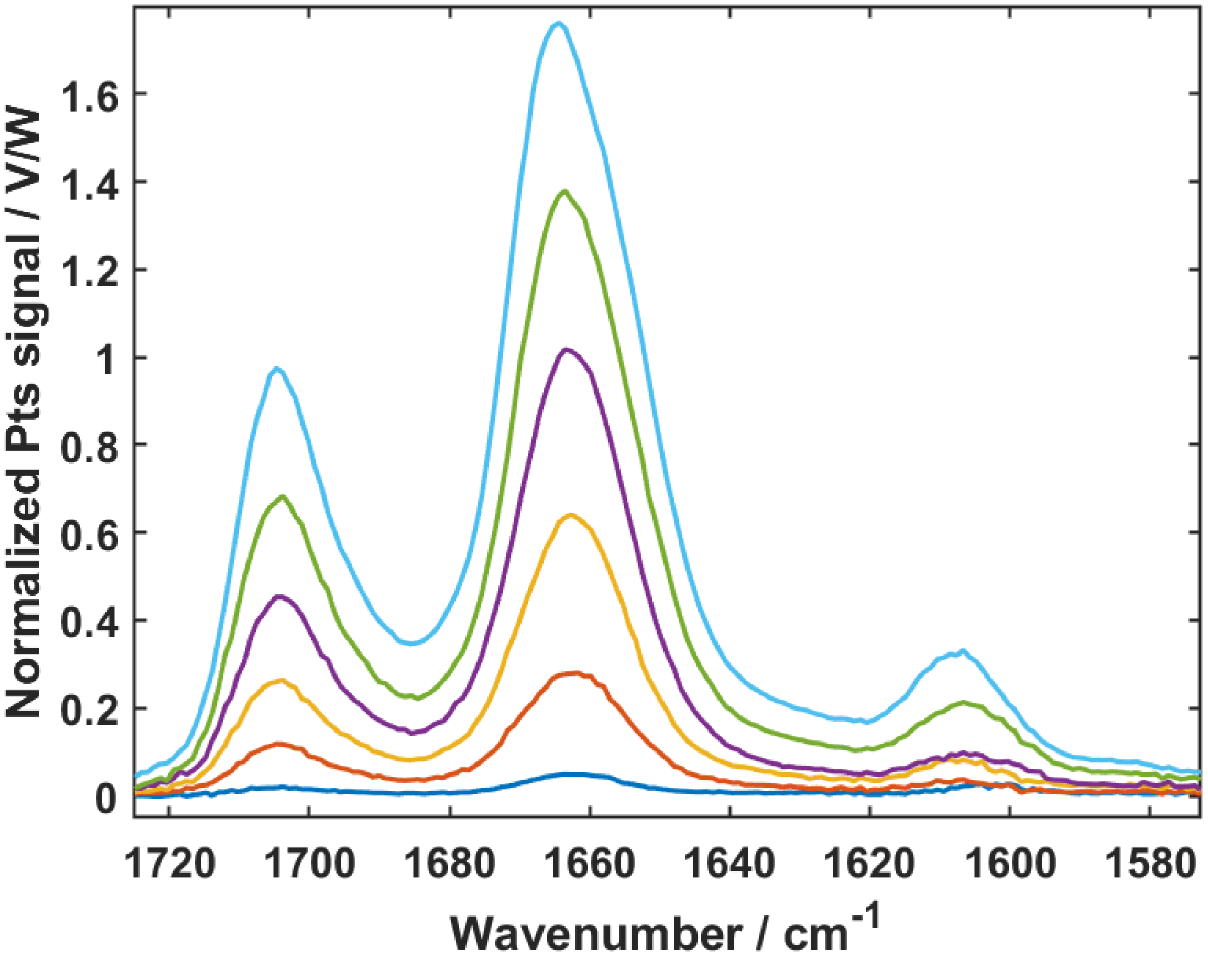

Figure S5. Unfiltered Normalized PTS signal.

As one can notice, the first caffeine peak from left-side appears, after normalization, higher in intensity. This happens because, as already mentioned, the optical power spectrum of the EC-QCL is wavelength-dependent meaning that at the sides of the spectrum, the available optical power is reduced in comparison to the central region where, for instance, the second caffeine peak is located.

Due to water vapor artifact/noise features still present in the spectra, a Savitzky-Golay filter (order: 3, window: 15 points / ~14 cm^{-1}) was used. The filter order and window length were chosen such that signal amplitudes/shapes are not affected, as reported below:

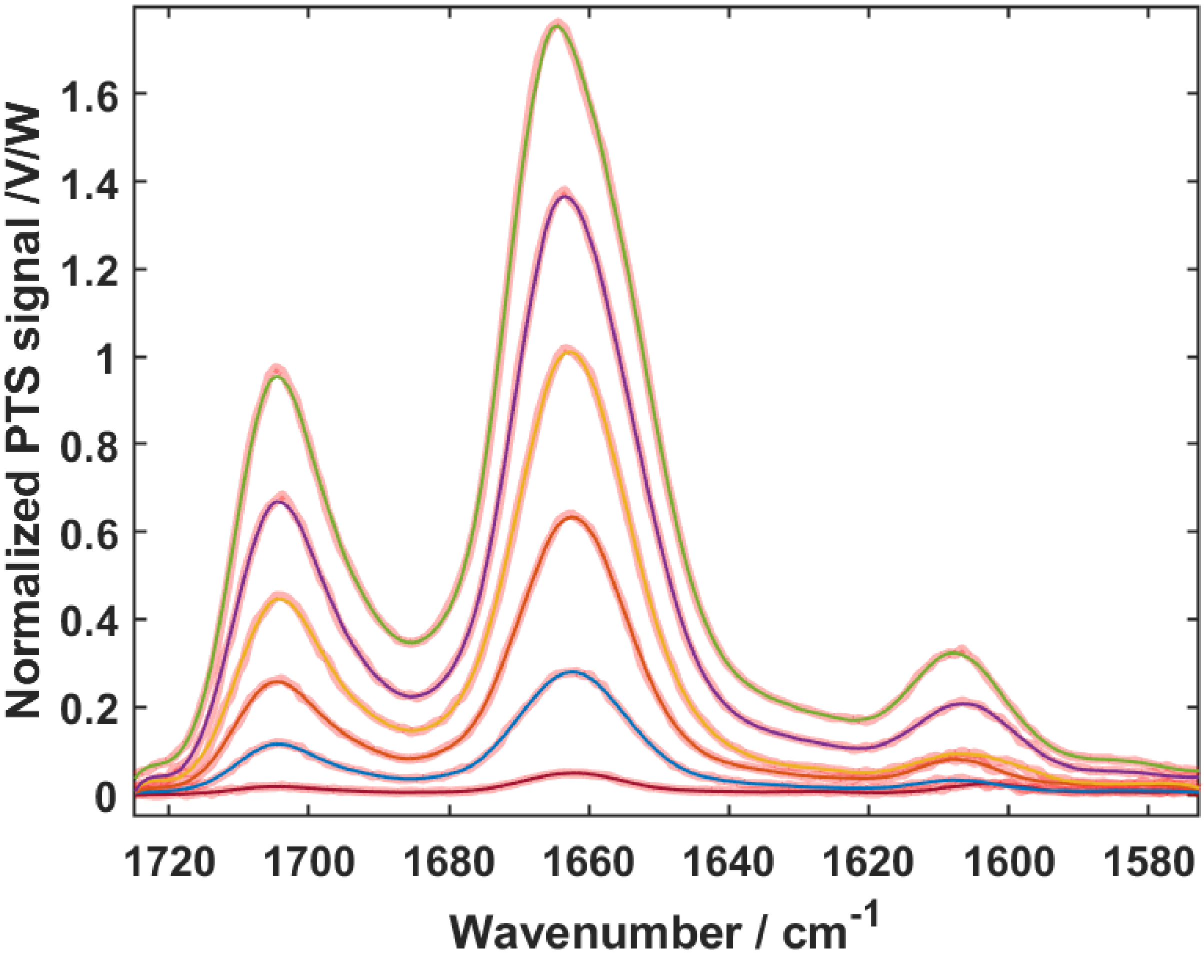

Figure S6. Filtered (solid lines) VS Unfiltered Normalized (opaque lines in red) PTS signal.

Error bars and uncertainties

In FTIR, absorbance spectra were recorded with OPUS 8.5.29 software package from Bruker. 10 spectra per each concentration were saved, with an integration time of 90s. The standard deviation of the 10 absorbance spectra was calculated at the band maximum at ~1660 cm^{-1} per each concentration. The computed values are reported in the form of error bars in Figure 3(b) and in the following table:

Table S1. Standard deviation at the spectrum absorption maximum for 10 replicates of each sample's concentration for FTIR

Caffeine Concentration [mg/mL]	Signal [-]	Standard deviation σ
0.1	0.02714	0.0003
0.5	0.12773	0.0002
1	0.25382	0.0001
1.5	0.39851	0.0001
2	0.51416	6.6e-05
2.5	0.6521	0.0001

For the PTS method, being the calibration fit performed on the normalized PTS signal, the following formula have been employed for estimation of the error bars reported in Figure 4(b). The standard

deviations have been evaluated on the 30 unfiltered replicates for each sample concentration, considering the std of the blank 30 unfiltered replicates, as follows:

$$\sigma_{(Normalized\ PTS\ signal)} = \sigma\left(\frac{I_{c\left[\frac{mg}{mL}\right]}[V] \cdot 1000\ - I_{blank}\ [V] \cdot 1000}{P(\tilde{\nu})[mW]}\right)$$

$$= \frac{1000}{P(\widetilde{\nu_{MAX}})}\sqrt{\left(\sigma^2_{I_{c\left[\frac{mg}{mL}\right]}(\widetilde{\nu_{MAX}})} + \sigma^2{}_{I_{blank}(\widetilde{\nu_{MAX}})}\right)}$$

Numerical values are reported in the following table:

Table S2.Standard deviation at the spectrum absorption maximum for 10 replicates of each sample's concentration for PTS

**Caffeine Concentration [mg/mL]**	**Signal [V/W]**	**Standard deviation σ**
0.1	0.04888	0.007
0.5	0.27972	0.020
1	0.63305	0.024
1.5	1.01075	0.028
2	1.36598	0.034
2.5	1.75493	0.056

When comparing the two techniques, it must be said that in the FTIR, even if we selected an integration time of 90 seconds to be comparable to PTS acquisitions time, the algorithm utilized by Bruker OPUS software will perform ~ 200 scans for obtaining a 90 second integration time. Meaning that the number of averaged scans in the FTIR evaluation is much higher than in the PTS spectrometer. This results in much lower uncertainties in the reference method.

100% lines evaluation for LOD/LOQ

The noise level, in this case, was calculated in the full spectral region between 1725 and 1570 cm^{-1} and used for the estimation of LOD and LOQ (equations (1) and (2) in the main text of the manuscript) both for FTIR and PTS spectrometers.

In the FTIR, 100% lines from two consecutively recorded measurements of the blank (chloroform in the transmission cell with a pathlength of 330 µm) were computed. 10 replicates (single channel acquisitions) were acquired in total, using again an integration time of 90 seconds.

The 10 replicates were paired for computation of 100% lines absorbance. 5 standard deviations were obtained and averaged. A mean σ of 1.9×10^{-4} was found.

In the PTS spectrometer 30 replicates x 3 seconds/scan for the background set were acquired 2 times. We followed the following procedure for estimating 100% lines in our system: each set of 30 scans was averaged, the difference between both averages was performed, then the resulting spectrum was multiplied by 1000, divided by the optical power of the excitation laser source and smoothed as when performing a PTS measurement (see "Data Acquisition and Processing" section). The noise level σ was found to be 3.6×10^{-4} V/W.

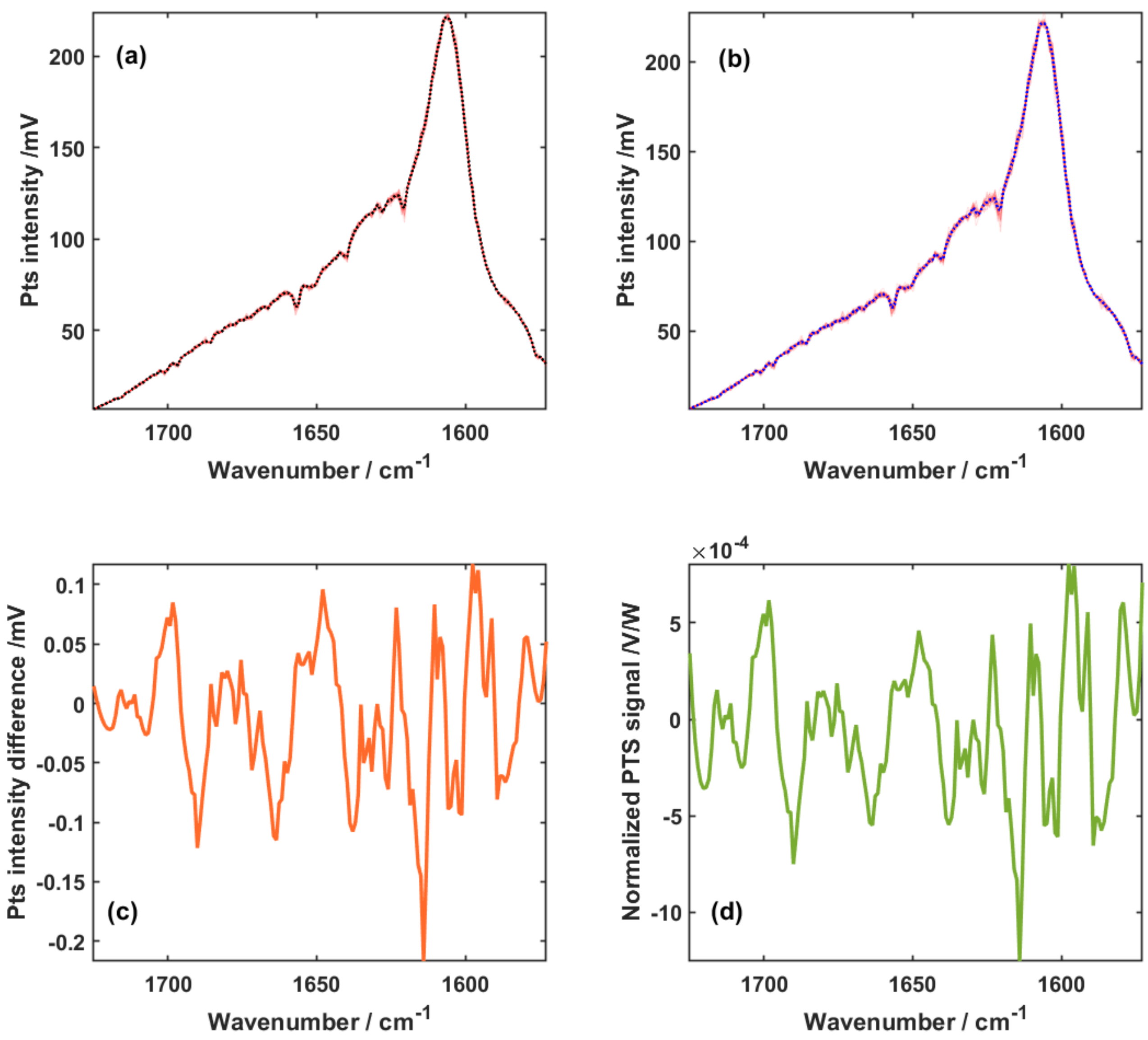

Figure S7. (a) Blank 30 replicates set 1 (red) and mean 1 value (black dots), set 1. (b) Blank 30 replicates set 2 (red) and mean 2 value (blue dots), set 2. (c) Difference between mean 1 and mean 2. (d) Normalization of (mean1-mean2) by optical power of EC-QCL
